# Clinical relevance of screening checklists for detecting cancer predisposition syndromes in Asian childhood tumours

**DOI:** 10.1038/s41525-018-0070-7

**Published:** 2018-11-15

**Authors:** Sock Hoai Chan, Winston Chew, Nur Diana Binte Ishak, Weng Khong Lim, Shao-Tzu Li, Sheng Hui Tan, Jing Xian Teo, Tarryn Shaw, Kenneth Chang, Yong Chen, Prasad Iyer, Enrica Ee Kar Tan, Michaela Su-Fern Seng, Mei Yoke Chan, Ah Moy Tan, Sharon Yin Yee Low, Shui Yen Soh, Amos Hong Pheng Loh, Joanne Ngeow

**Affiliations:** 1 Cancer Genetics Service, Division of Medical Oncology, National Cancer Centre Singapore, Singapore, 169610 Singapore; 2 SingHealth Duke-NUS Institute of Precision Medicine (PRISM), Singapore, 169856 Singapore; 3 VIVA-KKH Paediatric Brain and Solid Tumour Programme, KK Women’s and Children’s Hospital, Singapore, 229899 Singapore; 4 Department of Pathology and Laboratory Medicine, KK Women’s and Children’s Hospital, Singapore, 229899 Singapore; 5 Department of Paediatric Surgery, KK Women’s and Children’s Hospital, Singapore, 229899 Singapore; 6 Paediatric Hematology/Oncology Service, KK Women’s and Children’s Hospital, Singapore, 229899 Singapore; 7 Department of Neurosurgery, National Neuroscience Institute, Singapore, 308433 Singapore; 8 SingHealth Duke-NUS Neuroscience Academic Clinical Program, Singapore, 308433 Singapore; 9 Oncology Academic Clinical Program, Duke-NUS Medical School, Singapore, 169857 Singapore; 10 Lee Kong Chian School of Medicine, Nanyang Technological University, Singapore, 308232 Singapore; 11 Institute of Molecular and Cellular Biology, A*STAR, Singapore, 138673 Singapore

## Abstract

Assessment of cancer predisposition syndromes (CPS) in childhood tumours is challenging to paediatric oncologists due to inconsistent recognizable clinical phenotypes and family histories, especially in cohorts with unknown prevalence of germline mutations. Screening checklists were developed to facilitate CPS detection in paediatric patients; however, their clinical value have yet been validated. Our study aims to assess the utility of clinical screening checklists validated by genetic sequencing in an Asian cohort of childhood tumours. We evaluated 102 patients under age 18 years recruited over a period of 31 months. Patient records were reviewed against two published checklists and germline mutations in 100 cancer-associated genes were profiled through a combination of whole-exome sequencing and multiplex ligation-dependent probe amplification on blood-derived genomic DNA. Pathogenic germline mutations were identified in ten (10%) patients across six known cancer predisposition genes: *TP53, DICER1, NF1, FH, SDHD* and *VHL*. Fifty-four (53%) patients screened positive on both checklists, including all ten pathogenic germline carriers. *TP53* was most frequently mutated, affecting five children with adrenocortical carcinoma, sarcomas and diffuse astrocytoma. Disparity in prevalence of germline mutations across tumour types suggested variable genetic susceptibility and implied potential contribution of novel susceptibility genes. Only five (50%) children with pathogenic germline mutations had a family history of cancer. We conclude that CPS screening checklists are adequately sensitive to detect at-risk children and are relevant for clinical application. In addition, our study showed that 10% of Asian paediatric solid tumours have a heritable component, consistent with other populations.

## Cancer: Screening checklists catch Asian children at risk of hereditary tumours

Existing checklists designed to screen children for a genetic predisposition to cancer are sensitive enough to detect at-risk individuals in an Asian population. Joanne Ngeow from the National Cancer Centre Singapore and colleagues reviewed the medical records of 102 paediatric patients diagnosed with cancer and profiled for inherited mutations in 100 cancer-associated genes. They crosschecked the records against two published screening instruments used by clinical geneticists to identify patients with a hereditary cancer risk. Although only ten patients harboured known cancer predisposition mutations, the checklists flagged 54 individuals. However, all ten pathogenic gene carriers screened positive on the diagnostic aids. The checklists thus seem to err on the side of over-identification and don’t miss many true cases of hereditary cancer in this Asian population.

## Introduction

Genetic predisposition has been estimated to account for 4–10% of childhood cancers.^1,2^ However, recent genomic studies of paediatric cancer patients have suggested that 6–35% of children with cancer may harbour deleterious germline mutations associated with their disease,^3–7^ implying an underestimation of cancer predisposition syndrome (CPS) prevalence in the paediatric population. Identifying children with CPS has important clinical consequences for both patient and their family. First, diagnosis of CPS may facilitate decisions in clinical care such as modifying treatment plans to mitigate toxicity, initiating surveillance for early detection and intervention of secondary malignancies, or introducing targeted therapies.^8,9^ Second, family members identified to harbour the same germline mutation can be informed of their individual cancer risks, whereby appropriate cancer risk management and reproductive counselling can be provided.

However, for each child diagnosed with cancer, risk assessment for CPS has been, and remains, a tremendous challenge to the paediatric oncologist. Unlike adult cancers, age-of-onset is an unreliable indicator for CPS in paediatric patients. Furthermore, detection of CPS in children is complicated by the diverse and inconsistent presentations of recognizable clinical phenotypes and lack of clear associated family history.^3,8^ Therefore, the conundrum faced by most paediatric oncologists is navigating these complexities to identify at-risk children who will benefit from genetic testing and counselling. Several studies have attempted to assess risk factors that could reliably select for these patients.^8,10–13^ Overall, guidelines proposed through comprehensive expert panel reviews reveal several common criteria for recognizing children with CPS: specific neoplasms, medical/physical anomalies, family history and excessive toxicity of cancer therapy. These criteria are assembled in checklists to help paediatric oncologists screen for patients with CPS.

Most genomic profiling and clinical screening studies for paediatric cancer susceptibility are conducted on Caucasian populations. It is thus unknown whether their findings can extrapolate to Asian children. In this study, we aim to validate the utility of published checklists for screening children with CPS and in parallel, characterize the prevalence and spectrum of germline mutations in Asian patients with paediatric solid tumours through next-generation sequencing. We screened our prospectively enrolled cohort using two published clinical tools,^12,13^ and concurrently identified germline mutations in cancer predisposition genes using whole-exome sequencing and digital multiplex ligation-dependent probe amplification (MLPA). For a comprehensive evaluation of the genetic alterations, we further validated the somatic status of identified germline variants in prospectively collected patient tumours.

## Results

### Patient characteristics

**Fig. 1:**
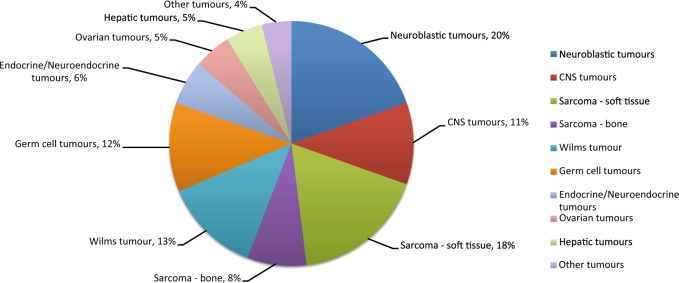
Distribution of tumour diagnoses included in this study. CNS central nervous system

Our study included 102 children under age 18 years enrolled between January 2015 and August 2017. Patient characteristics and demographics are summarized in Table 1. According to national registry data, our cohort represents over 80% of all malignant paediatric tumours in Singapore.^14,15^ Fifty-two boys and 50 girls were included, with a median age at diagnosis of 4 years. Ethnic distribution was reflective of our population, comprising predominantly Chinese, followed by Indian, Malay and other ethnicities. The children presented a wide spectrum of solid tumours, broadly classified into ten histological groups (Fig. 1). Neuroblastic tumours are the most commonly observed, accounting for 19.6% (*n* = 20) of all solid tumours, followed by central nervous system (CNS) tumours in 11 (10.8%) children. A total of 26 (25.4%) patients presented with soft tissue and bone sarcomas of various histological classifications whereas 13 (12.7%) had Wilms tumour and 12 (11.8%) patients with extracranial germ cell tumours. The remaining children had neoplasms broadly grouped as endocrine or neuroendocrine (*n* = 6), ovarian (*n* = 5), hepatic (*n* = 5), and other rare tumours (*n* = 4), including pleuropulmonary blastoma (PPB, *n* = 1), nasopharyngeal carcinoma (*n* = 1) and Langerhans cell histiocytosis (*n* = 2) (Supplementary Table 1).

**Table 1:** Characteristics of 102 childhood tumour patients

Characteristics	No. (%)
Demographics	
Age at diagnosis, median (IQR), years	4 (2–12)
<1 year	12 (11.8)
1–5 years	45 (44.1)
6–10 years	13 (12.7)
11–19 years	32 (31.4)
Gender	
Male	52 (51.0)
Female	50 (49.0)
Ethnicity	
Chinese	62 (60.8)
Indian	13 (12.7)
Malay	9 (8.8)
Other	18 (17.6)

*IQR* interquartile range

### Clinical checklist-aided screening for cancer predisposition syndrome

**Fig. 2:**
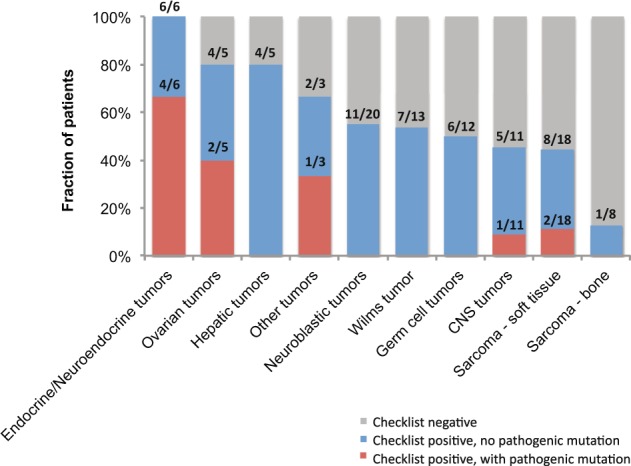
Clinical checklist screening outcomes and germline mutation frequencies among evaluated patients. Proportion of checklist-positive screenings and pathogenic germline mutation carriers are indicated above each bar

Using two published clinical checklists, we assessed 101 patients in our cohort for CPS. One patient was not evaluable due to insufficient clinical data. We found 79 (77.5%) and 66 (64.7%) patients with clinical features indicative of CPS based on the criteria proposed by Ripperger et al. or Postema et al. respectively,^11–13^ whereas ten (9.8%) patients were negative for both assessments (Table 2). Of the patients who screened positive, 54 (52.9%) met criteria in both checklists. Analysis of this group by tumour type revealed that patients with endocrine or neuroendocrine tumours are most likely to screen positive, while sarcoma patients were least likely to meet the criteria (Fig. 2). Both checklists were equally sensitive in detecting all ten patients with pathogenic germline mutations identified by sequencing (Table 2). While the use of each checklist independently was less specific, combining criteria of both checklists improved the specificity of detection to 52% without effect on sensitivity.

**Table 2:** Sensitivity and specificity of the two assessed clinical screening checklists

Measure	Checklist		
	By Ripperger et al.	By Postema et al.	In combination
Checklist screening outcome, No. (%)			
Not evaluated^a^	1 (1.0)	1 (1.0)	1 (1.0)
Evaluated positive	79 (77.5)	66 (64.7)	54 (52.9)
Evaluated negative	22 (21.5)	35 (34.3)	10 (9.8)
Evaluation outcome^b^, No. (%)			
Checklist positive with pathogenic germline mutation	10 (9.9)	10 (9.9)	10 (9.9)
Checklist positive without pathogenic germline mutation	69 (68.3)	56 (55.4)	44 (43.6)
Checklist negative with pathogenic germline mutation	0	0	0
Checklist negative without pathogenic germline mutation	22 (21.8)	35 (34.7)	47 (46.5)
Checklist assessment (%)			
Sensitivity	100	100	100
Specificity	24	38	52

^a^Patient excluded due to incomplete clinical data

^b^Calculation excluded patient not evaluated on checklist

Of the ten patients who screened positive and carried pathogenic germline mutations, eight fulfilled clinical criteria for CPS including Li-Fraumeni syndrome (LFS, *n* = 3), *DICER1* syndrome (*n* = 3), neurofibromatosis type 1 (NF1) (*n* = 1) and von Hippel-Lindau (VHL, *n* = 1) syndrome (Table 3). Nine patients were referred by their primary oncologist for genetic counselling and testing, of which four did not follow up with the recommendation. One patient was not referred due to rapid disease progression and succumbed to complications arising from her condition.

**Table 3:** List of patients with identified pathogenic germline mutations

Subject ID	Sex	Age at diagnosis (yr)	Diagnosis	Clinical features	Family history	Clinical (CPS) diagnosis	Referred for genetic counselling by primary physician?	Ripperger et al. criteria	Postema et al. criteria	Gene	Mutation type	DNA change^a^	Protein change	ACMG Classification	Variant inheritance status	Somatic LOH	Somatic second mutation
DR615	M	2.6	DA	Multiple café-au-lait spots Plexiform neurofibroma Axillary and inguinal freckling Global developmental delay Frequent seizures	N	NF1	Y^b^	+	+	*NF1*	Nonsense	c.4537 C > T	p.Arg1513*	P	ND	N	N
										*TP53*	Gross deletion	Deletion of exon 1	Nil	LP	ND	Y	N
N074	M	11.8	PC	Drop in growth rate from 50th percentile to 10th percentile	N	Nil	Y	+	+	*SDHD*	Frameshift insertion	c.10dupC	p.Leu4Profs*65	P	ND	ND	ND
DR552	F	7.3	PC	Ocular papilloedema	Mother: PC Maternal SDR: RCC, SLE/RA	VHL syndrome	Y	+	+	*VHL*	Missense	c.191G > C	p.Arg64Pro	P	ND	Y	ND
DR678	F	1.2	ACC	Hirutism Clithoromegaly	Unremarkable^c^	LFS	Y	+	+	*TP53*	Missense	c.638G > A	p.Arg213Gln	P	Mat	Y	ND
DR383	F	14.6	ACC	Mild hirutism Elevated male hormones Late puberty	Father: pancreatic ca.	LFS	Y^b^	+	+	*TP53*	Missense	c.422G > A	p.Cys141Tyr	P	ND	Y	N
DR623	F	14.2	SLCT	MNG Hirutism Precocious puberty	Mother: MNG (21 y), ovarian cysts, *DICER1* mutation carrier Sister: Thyroid ca. (12 y) Brother: MNG	*DICER1* syndrome	Y^b^	+	+	*FH*	Nonsense	c.32C > A	p.Ser11*	P	ND	Y	N
										*DICER1*	Frameshift insertion	c.2281dupT	p.Tyr761Leufs*5	P	Mat	N	*DICER1*: c.5437G > A (p.Glu1813Lys)
DR656	F	6.9	SLCT	Thyroid nodules Precocious puberty	Mother: Thyroid nodules Maternal SDR: Thyroid nodules, uterine ca. Paternal SDR: Leukaemia, breast ca. Paternal TDR: NPC	*DICER1* syndrome	Y^b^	+	+	*DICER1*	Frameshift insertion	c.4085dupA	p.Lys1362Glufs*13	P	ND	N	*DICER1*: c.5438A > G (p.Glu1813Gly)
DR673	F	4.7	PPB	Acute respiratory distress	Unremarkable^c^	*DICER1* syndrome	N	+	+	*DICER1*	Frameshift deletion	c.4405_4406del	p.Leu1469Phefs*7	P	ND	N	*DICER1*: c.5439G > T (p.Glu1813Asp)
N096	M	0.0	RMS	Nil	Paternal SDR: Leukaemia (18 y), breast ca. (28 y), NSCLC (69 y) Maternal TDR: NPC (75 y)	LFS	Y	+	+	*TP53*	Missense	c.329G > C	p.Arg110Pro	P	Pat	Y	ND
N431	F	17.6	LPS	Nil	Mother: breast cysts (34 y) Father: benign ear tumour (10 y), SCC of scrotum (50 y), *TP53* mutation carrier Maternal SDR: uterine ca. (40 y), tongue ca. (55 y)^c^	Nil	Y	+	+	*TP53*	Missense	c.817C > T	p.Arg273Cys	P	Pat	ND	ND

*ACC* adrenocortical carcinoma, *CPS* cancer predisposition syndrome, *DA* diffuse astrocytoma, *F* female, *LFS* Li−Fraumeni syndrome, *LP* Likely pathogenic, *LPS* liposarcoma, *M* male, *Mat* maternally-inherited, *MNG* multinodular goitre, *N* No, *ND* not determined, *NF1* neurofibromatosis 1, *NPC* nasopharyngeal cancer, *NSCLC* non-small cell lung cancer, *P* pathogenic, *Pat* paternally inherited, *PC* pheochromocytoma, *PPB* pleuropulmonary blastoma, *RMS* rhabdomyosarcoma, *RCC* renal cell carcinoma, *SCC* squamous cell carcinoma, *SDR* second-degree relatives, *SLCT* Sertoli−Leydig cell tumour, *SLE/RA* systemic lupus erythematosus/rheumatoid arthritis, *TDR* third-degree relatives, *VHL* von Hippel-Lindau, *Y* yes

^a^Zygosity status of all germline mutations was heterozygous

^b^Patient was referred for genetic counselling but failed to follow up with the recommendation

^c^Family history recorded is not extensive

### Spectrum of germline mutations in known cancer predisposition genes

**Fig. 3:**
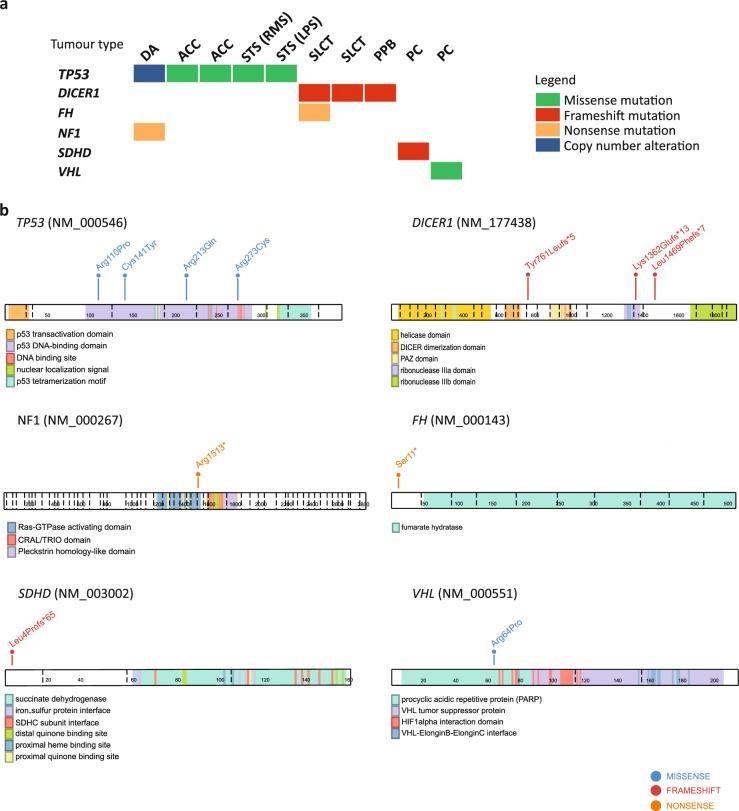
Prevalence of pathogenic germline mutations. **a** Overview of identified pathogenic germline mutations across six genes in ten patients. **b** Lollipop diagrams visually depicting occurrence of the pathogenic germline mutations on proteins encoded by the affected genes. DA diffuse astrocytoma, ACC adrenocortical carcinoma, STS soft tissue sarcoma, RMS rhabdomyosarcoma, LPS liposarcoma, SLCT Sertoli−Leydig cell tumour, PPB pleuropulmonary blastoma, PC pheochromocytoma

We identified 12 pathogenic germline mutations in 10 children (9.8%) across six known cancer predisposition genes (Table 3). Frequency of mutations was highest in *TP53* affecting five patients, followed by *DICER1* mutations in three patients (Fig. 3a). Two patients harboured more than one pathogenic variant: one with diffuse astrocytoma was found to have concurrent *TP53* exon 1 deletion and *NF1* nonsense mutation, and a Sertoli−Leydig cell tumour (SLCT) patient harboured both *DICER1* frameshift and nonsense *FH* mutations. In addition, two patients were found with a deleterious mutation in *VHL* and *SDHD* respectively. Pathogenic variants in autosomal recessive genes were not observed.

All *TP53* variants identified were previously seen in LFS families.^16–20^ Four of these were missense mutations that clustered within the p53 DNA-binding domain (Fig. 3b). Three (Cys141Tyr, Arg213Gln, Arg273Cys) are known to reduce p53 transactivation activity^21,22^, whereas Arg110Pro was demonstrated to have a dominant-negative effect on wild-type p53 function.^23^ Somatic loss-of-heterozygosity (LOH) was observed in all *TP53* mutation carriers, including the hemizygous exon 1 deletion carrier. *TP53* exon 1 encodes the 5′ untranslated-region (UTR) shown to be critical for RPL26-mediated translation of p53 mRNA upon DNA damage.^24^ Reported carriers of *TP53* promoter or exon 1 deletion mostly presented soft tissue sarcomas and breast cancer.^20,25–27^ Somatic LOH reflected by homozygous deletion of this region in our patient’s tumour (Supplementary Figure 1) implicates deleterious effect of this variation. Interestingly, this child also harboured a truncating germline mutation in *NF1* (Arg1513*) previously observed in other NF1 patients.^28–30^

The three detected *DICER1* germline mutations were truncating variants, two of which are reported in ClinVar database. A second *DICER1* somatic mutation was found in all three patients at the RNase IIIb domain hotspot Glu1813 residue known to inactivate DICER1 activity^31,32^ (Fig. 3b). A concurrent truncating *FH* mutation with somatic LOH was seen in one of the *DICER1* germline mutation carriers (Table 3).

Amongst the tumour types, prevalence of pathogenic germline mutation was highest in endocrine or neuroendocrine tumours (*n* = 4/6, 67%). This is followed by ovarian tumour (*n* = 2/5, 40%), soft tissue sarcoma (*n* = 2/18, 11%) and CNS tumour (*n* = 1/11, 9%) (Fig. 2). Further breakdown by histological subtypes highlighted that all enrolled patients with adrenocortical carcinoma (ACC), pheochromocytoma and SLCT harboured germline mutations, implying a higher genetic susceptibility in these tumour types (Supplementary Table 2).

### Association of germline mutations with clinical phenotype and family history

Overall, the detected genotypes were consistent with patient clinical phenotypes. Four among five *TP53* mutation carriers presented tumours typical of LFS spectrum: ACC (*n* = 2) and soft tissue sarcoma (*n* = 2) (Fig. 3a). Moreover, their mutations were also reported in LFS families and paediatric patients with similar tumour types.^3,16–18,33^ Similarly, germline mutations in *SDHD* and *VHL*, two genes known to confer susceptibility to pheochromocytoma, were found in both patients with this diagnosis (Fig. 3a). Unsurprisingly, our *DICER1* germline mutation carriers presented tumour types most frequently associated with *DICER1* syndrome, namely PPB (*n* = 1) and SLCT with multinodular goitre (MNG) (*n* = 2).^32^

In patients harbouring more than one pathogenic germline mutation, clinical manifestations were predominantly consistent with genes in which penetrance is greater at an earlier age. For instance, the 2.6-year-old diffuse astrocytoma patient with concurrent *NF1* and *TP53* mutations exhibited clinical features mostly characteristic of NF1: multiple café-au-lait spots, neurofibroma and global developmental delay. This is congruent with the fact that penetrance is almost 100% by age 8 years in *NF1* germline mutation carriers^34^ compared to penetrance of 50% by the third decade of life in individuals with germline *TP53* mutations.^35^ However, the presence of astrocytoma unrelated to the optic pathway at this age is more consistent with the germline *TP53* mutation harboured by this child, which was missed by the treating physicians given his characteristic NF1 clinical features.

Six of the ten germline mutation carriers had at least one relative with cancer (Table 3); however, only five (50%) showed a family history typical of CPS. Two are *TP53* mutation carriers, who met Chompret criteria for LFS, whereas two *DICER1* mutation carriers with SLCT had multiple relatives presenting MNG and thyroid cancer. The remaining *VHL* mutation carrier demonstrated a family history of pheochromocytoma and renal cell carcinoma consistent with VHL syndrome.

### Variants of uncertain significance in known cancer predisposition genes

A total of 86 rare variants of uncertain significance (VUS) were detected, predominantly in neuroblastic tumours with an adjusted frequency of four variants, followed by soft tissue sarcoma with three VUS (Supplementary Figure 1). Amongst these, some were predicted potentially deleterious by in silico algorithms and occurred in DNA repair genes, including *BRCA1, BRCA2, MLH1, PMS2* and *RAD54L*. Coincidentally, tumours of patients from these two histological subtypes exhibited higher incidences of karyotypic aberrations, suggesting plausible roles for DNA repair pathway deficiency.

A few potentially deleterious VUS were found in genes beyond those commonly associated with the disease. For example, a variant in *MAX*, an essential interacting partner of MYC, was identified in a patient with neuroblastoma. The mutation Arg100Cys, which occurred within the leucine-zipper domain of MAX important for regulation of MYC, could impair MYC activities. Additionally, we found two variants—*CDH1* Pro373Leu and *RAF1* Pro332Ala—individually associated with hereditary diffuse gastric cancer (HDGC)^36^ and childhood-onset dilated cardiomyopathy^37^ in two patients with hepatocellular carcinoma (HCC) and testicular germ cell tumour respectively. Although demonstrated to be functionally deleterious, association of these VUS with the clinical phenotype of our patients remains to be verified by further studies.

## Discussion

Although genomic sequencing has expanded our understanding of paediatric cancer predisposition and presented opportunities for genetics-mediated care, identifying children at-risk remains a clinical challenge for paediatric oncologists. Expert panels have deliberated over clinical and genetic attributes of multitude predisposition syndromes and assembled checklists aimed at facilitating detection of these children. Our study evaluated two such clinical tools in an Asian cohort of paediatric solid tumours and found both sufficiently sensitive for identifying at-risk children. Combining the criteria of both checklists saw a marked improvement in specificity, implicating possibility of increasing stringency in evaluation without compromising sensitivity of screening.

Our data reflected disparity in the yield of germline mutation carriers compared to clinically positive screenings (Fig. 2), implying that utility of the clinical checklists might vary by tumour types. This discordance could be attributed to several factors. First, our data together with other studies demonstrated variable prevalence of genetic susceptibility in different paediatric tumours, ranging from <5% in neuroblastoma and Wilms tumour to over 50% in ACC and pheochromocytoma.^3,38–40^ Second, while tumours such as ACC are strongly correlated with well-known susceptibility genes and CPS, e.g. *TP53* and LFS, genetic alterations in tumours that are associated with little-known CPS or susceptibility genes are likely underestimated. Thus, lack of detectable germline mutations in subtypes such as neuroblastic tumours may be attributed to alterations in genes and CPS beyond the currently defined spectrum investigated. Hence, while our study demonstrated clinical relevance of these checklists, it cautioned for careful tumour type-specific considerations in application of this tool and more importantly, highlights the need for further research into novel susceptibility genes associated with childhood tumours.

Prevalence of pathogenic germline mutations identifiable by next-generation sequencing in our Asian cohort is 9.8%, consistent with the range of 8–10% observed in other studies.^3,5,7^ This is despite exclusion of haematologic malignancies and a lower incidence of CNS tumours, which were previously reported with a greater than average prevalence of germline mutations.^4^ Taken together, our study confirms that genetic predisposition accounts for approximately 10% of all childhood solid tumours, which is consistent with the prevalence of 8% observed in adult cancers.^41^

Nevertheless, this prevalence in genetic susceptibility is potentially a conservative estimate limited to our current knowledge of CPS and spectrum of associated genes. Approximately 50% of our patients who screened positive either harboured a VUS or had clinical features strongly suggestive of CPS. For instance, the germline mutation *CDH1* Pro373Leu previously identified in an HDGC family and shown to impair E-cadherin in vitro^36^ was detected in our HCC patient. Although presently classified as a VUS due to insufficient evidence for its role in HCC tumourigenesis, it is imperative that variants of uncertain significance are periodically reviewed as new research uncovers novel genotype−phenotype associations.

Presentation of unusual neoplasms for a diagnosed CPS may stem from multiple pathogenic germline mutations, as demonstrated in our NF1 patient presenting with diffuse astrocytoma at age 2.6 years who was subsequently found to also harbour a pathogenic germline *TP53* mutation. Children with NF1 are predisposed to CNS tumours typically of pilocytic astrocytoma subtype in the optic pathway, often accompanied by complete inactivation of *NF1* through somatic events.^42^ Grade II gliomas such as diffuse astrocytoma are uncommon in paediatric patients and more frequently associated with *TP53* inactivation.^43^ Intriguingly, somatic *TP53* LOH, but not *NF1*, was observed in our patient’s tumour. Deficiency of p53 prior to NF1 loss has been correlated with complete penetrance of malignant astrocytomas in mice^44^ and could explain the histological subtype presented by our patient. Also noteworthy is that deletion of the *TP53* exon 1 in this patient was not detected on whole-exome sequencing but identified through MLPA, hence would have been also missed by next-generation sequencing panels currently utilized for clinical genetic testing. This demonstrates the limitations of next-generation sequencing panels and highlights the need to include comprehensive capture of larger copy number alterations as well as untranslated gene regions in clinical genetic testing.

As with similar studies on germline variation in cancer,^3,41^ the rarity of paediatric solid tumours and diverse histological subtypes in this study precluded statistical significance in observed associations. Furthermore, detection of pathogenic germline mutations is limited to known cancer predisposition genes. Genetically unresolved cases may have pathogenic germline mutations in novel predisposition genes^5^ and data on VUS from our study highlights the particular tumour types that could benefit from further research into novel cancer predisposition genes.

In conclusion, our study validated two clinical checklists for detection of children at risk of CPS, and demonstrated that heritable predisposition accounts for at least 10% of Asian paediatric solid tumours. Application of these checklists is expected to improve identification of children at risk of CPS and referral for genetic testing, with significant implications on treatment strategies and clinical care for paediatric solid tumour patients and their families.

## Materials and methods

### Patients and specimens

Patients consulted at our paediatric oncology clinic at KK Women’s and Children’s Hospital and the Cancer Genetics Service at National Cancer Centre Singapore were prospectively recruited for this study. In all, 102 patients under age 18 years of various solid tumour types were included. Data on clinical history, tumour histology, treatment modalities and family history of cancer were collated. Collected peripheral blood and excess tissues from routine tumour biopsies or resections were stored at −80 °C. All tumour specimens were evaluated by a consultant pathologist to be representative lesional tissue on frozen section histology. This study was approved by SingHealth Centralised Institutional Review Board (IRB 2018/2456, 2014/2079) with signed informed consent from patients and guardians.

### Cancer predisposition syndrome screening checklist

To validate the utility of clinical checklists for screening paediatric patients at risk of CPS, collated clinical data were assessed independently against two published guidelines.^11–13^ Broadly, criteria outlined in the two checklists for consideration included family history of cancer, aberrant tumour genetics, multiple malignancies in the index patient, congenital defects, excessive toxicity related to cancer therapy, and specific tumour types that have been reported to frequently associate with syndromic disorders. Patient records were reviewed and given a positive score if one or more criteria was fulfilled for each checklist.^12,13^

### Whole-exome sequencing

Genomic DNA from blood and tissue specimens were extracted using QIAamp DNA mini kit (Qiagen, Hilden, Germany) according to the manufacturer’s protocol. Purified DNA was sheared to 200 base pairs (bp) and exome captured using Agilent SureSelect V6 kit. Constructed libraries were sequenced on Illumina Hiseq4000 (Illumina, San Diego, CA, USA) to an average depth of 72× with over 92% of target bases covered >20×.

### Variant prioritization pipeline

Sequenced reads were aligned to the human reference genome (hs37d5) using Burrows−Wheeler Aligner (BWA) and variants called using Freebayes, as detailed under Supplementary Methods. Variants were filtered by read depth (10×) and quality score (Phred score > 30), annotated using ANNOVAR and curated in a stepwise manner into five classifications: pathogenic, likely pathogenic, VUS, likely benign, benign. To identify candidate variants in autosomal dominant cancer predisposition genes (Supplementary Table 3), we filtered for rare coding and splice-site variants, determined by a minor allele frequency (MAF) of ≤0.1% in Exome Aggregation Consortium (ExAC), 1000 Genomes (1000G) databases and our in-house database of local population (*n* = 1412). Truncating, splice-site and missense variants with a REVEL score of ≥0.6 and/or a Phred-scaled CADD score of ≥20 or without annotation were assessed for pathogenicity by the American College of Medical Genetics and Genomics (ACMG) guidelines.^45^ For autosomal recessive genes (Supplementary Table 3), we applied an MAF ≤5% threshold and curated only homozygous variants or two compound heterozygous variants within the same gene by ACMG guidelines. All other variants failing to meet criteria for benign/likely benign/likely pathogenic/pathogenic classifications by ACMG guidelines were classified as VUS. Protein domains were visualized using ProteinPaint.^46^

### Digital multiplex ligation-dependent probe amplification analysis

MLPA targeting 29 hereditary cancer genes (Supplementary Table 4) was performed on patient genomic DNA as previously described^27^ and data analysed in collaboration with the manufacturer using a pre-release version of Coffalyser.Net (MRC-Holland, Amsterdam, The Netherlands).

### Validation of variants

Candidate variants were validated by Sanger sequencing using BigDye Terminator v3.1 (ABI, ThermoFisher Scientific Corporation) and resulting chromatograms analysed using Mutation Surveyor (Softgenetics, PA, USA). Copy number variants detected through MLPA were validated by quantitative PCR. Cycle threshold (*C*_t_) values were normalized to GAPDH endogenous control and fold-change in gene dosage was calculated using the ΔΔ*C*_t_ method by normalizing against two healthy controls. Somatic status of variants was similarly validated on tumour DNA.

### Statistical analyses

Patient characteristics and sequencing results were tabulated with descriptive statistics including median, interquartile range and proportions.

## Electronic supplementary material

Supplementary Material

## Data Availability

All sequencing data from this study are publicly available at European Nucleotide Archive (accession PRJEB28383).

## Electronic supplementary material

**Supplementary information** accompanies the paper on the *npj Genomic Medicine* website (10.1038/s41525-018-0070-7).
