# Supplementary material for: Clinical relevance of screening checklists for detecting cancer predisposition syndromes in Asian childhood tumours
Source: NPJ Genom Med. 2018 Nov 15;3:30. doi: 10.1038/s41525-018-0070-7 (PMC6237849; doi:10.1038/s41525-018-0070-7)
Supplement: Supplementary file 1 — Supplementary Material [file 41525_2018_70_MOESM1_ESM.docx]

**Clinical Relevance of Screening Tools for Detection of Cancer Predisposition Syndromes in an Asian Cohort of Childhood Tumours**

**SUPPLEMENTARY INFORMATION**

**SUPPLEMENTARY METHODS**

**Variant prioritization pipeline**

Sequenced reads were aligned to human reference genome (hs37d5) using Burrows-Wheeler Aligner (BWA) and variants called using Freebayes, as detailed under Supplementary Methods. Variants were filtered by read depth (10X) and quality score (Phred score>30), then annotated using ANNOVAR (version 2016FEB01) and curated in a stepwise manner into five classifications: pathogenic, likely pathogenic, variant of uncertain significance (VUS), likely benign, benign. To identify candidate variants in autosomal dominant cancer predisposition genes (Supplementary Table 1), we filtered for rare coding and splice site variants, determined by a minor allele frequency (MAF) of ≤0.1% in Exome Aggregation Consortium (ExAC), 1000 Genomes (1000G) databases and our in-house database of local population (n=1412). Truncating, splice site and missense variants with a REVEL score of ≥ 0.6 and/or a phred-scaled CADD score of ≥20 or without annotation were assessed for pathogenicity by the American College of Medical Genetics and Genomics (ACMG) guidelines^14^. For autosome recessive genes (Supplementary Table 1), we filtered for variants with a MAF ≥ 5% in ExAC, 1000G and our in-house database, then assessed only homozygous variants or two compound heterozygous variants within the same gene in the patient by ACMG guidelines. Protein domains were visualized using ProteinPaint^15^.

**SUPPLEMENTARY TABLES**

Supplementary Table 1 : Tumour diagnoses and histological subtypes included in this study.

| **Tumour Type** | **Subtype** | **No. patients (%)** |
| --- | --- | --- |
| **Neuroblastic tumours** | | **20 (20%)** |
|  | Neuroblastoma | 13 |
|  | Ganglioneuroblastoma | 2 |
|  | Ganglioneuroma | 3 |
|  | Esthesioneuroblastoma | 1 |
|  | Schwannoma | 1 |
| **Central Nervous System (CNS) tumours** | | **11 (11%)** |
|  | Medulloblastoma group 4 | 3 |
|  | Medulloblastoma group 3 | 1 |
|  | Ependymoma | 2 |
|  | Diffuse intrinsic pontine glioma (DIPG) | 2 |
|  | Diffuse astrocytoma | 1 |
|  | Ganglioglioma | 1 |
|  | Diffuse leptomeningeal glioneuronal tumour (DL-GNT) | 1 |
| **Sarcoma - soft tissue** | | **18 (18%)** |
|  | Lipoblastoma | 3 |
|  | Rhabdomyosarcoma | 3 |
|  | Rhabdoid tumour | 3 |
|  | Angiomatoid fibrous histocytoma | 2 |
|  | Desmoid fibromatosis | 2 |
|  | Dermatofibrosarcoma protuberans (DFSP) | 1 |
|  | Alveolar soft-part Sarcoma (ASPS) | 1 |
|  | Liposarcoma | 1 |
|  | Synovial sarcoma | 1 |
|  | Benign spindle cell lesion | 1 |
| **Sarcoma - bone** |  | **8 (8%)** |
|  | Osteosarcoma | 4 |
|  | Ewing's sarcoma | 2 |
|  | Chondroblastoma | 1 |
|  | Chondrosarcoma | 1 |
| **Wilms tumour** |  | **13 (13%)** |
| **Germ cell tumours** |  | **12 (15%)** |
|  | Teratoma | 5 |
|  | Yolk sac tumour | 3 |
|  | Dysgerminoma | 2 |
|  | Mixed germ cell tumour | 2 |
| **Endocrine/Neuroendocrine tumours** | | **6 (6%)** |
|  | Adrencortical carcinoma (ACC) | 2 |
|  | Pheochromocytoma | 2 |
|  | Paraganglioma | 1 |
|  | Thyroid carcinoma | 1 |
| **Ovarian tumours** |  | **5 (4%)** |
|  | Sertoli-Leydig cell tumour (SLCT) | 2 |
|  | Juvenile granulosa ovarian cell tumour (JGOCT) | 2 |
|  | Mucinous ovarian tumour | 1 |
| **Hepatic tumours** |  | **5 (5%)** |
|  | Hepatoblastoma | 4 |
|  | Hepatocelluar carcinoma | 1 |
| **Other tumours** |  | **4 (4%)** |
|  | Langerhans cell histocytosis | 2 |
|  | Nasopharyngeal carcinoma | 1 |
|  | Pleuropulmonary blastoma (PPB) | 1 |
|  |  |  |

Supplementary Table 2 : Overview of identified pathogenic germline mutations across tumour histological subtypes.

| **Tumour type** | **Subtype** | **Total** | **Pathogenic mutation** | **No pathogenic mutation** |
| --- | --- | --- | --- | --- |
| **Endocrine/Neuroendocrine tumour** | | **6** | **4** | **2** |
|  | Adrenocortical carcinoma (ACC) | 2 | 2 | 0 |
|  | Pheochromocytoma | 2 | 2 | 0 |
|  | Paraganglioma | 1 | 0 | 1 |
|  | Thyroid carcinoma | 1 | 0 | 1 |
| **Ovarian tumour** | | **5** | **2** | **3** |
|  | Sertoli-Leydig cell tumour (SLCT) | 2 | 2 | 0 |
|  | Juvenile granulosa cell tumour | 2 | 0 | 2 |
|  | Mucinous ovarian tumour | 1 | 0 | 1 |
| **Other tumours** | | **3** | **1** | **2** |
|  | Pleuropulmonary blastoma (PPB) | 1 | 1 | 0 |
|  | Langerhans cell histiocytosis | 1 | 0 | 1 |
|  | Nasopharyngeal carcinoma | 1 | 0 | 1 |
| **Germ cell tumour (GCT)** | | **12** | **0** | **12** |
|  | Teratoma | 5 | 0 | 5 |
|  | Yolk sac tumour | 3 | 0 | 3 |
|  | Dysgerminoma | 2 | 0 | 2 |
|  | Mixed GCT | 2 | 0 | 2 |
| **Central nervous system (CNS) tumour** | | **11** | **1** | **10** |
|  | Diffuse astrocytoma | 1 | 1 | 0 |
|  | Medulloblastoma group 4 | 3 | 0 | 3 |
|  | Ependymoma | 2 | 0 | 2 |
|  | Diffuse intrinsic pontine glioma (DIPG) | 2 | 0 | 2 |
|  | Ganglioglioma | 1 | 0 | 1 |
|  | Medulloblastoma group 3 | 1 | 0 | 1 |
|  | Diffuse leptomeningeal glioneuronal tumour (DL-GNT) | 1 | 0 | 1 |
| **Sarcoma - soft tissue** | | **18** | **2** | **16** |
|  | Liposarcoma (LPS) | 1 | 1 | 0 |
|  | Rhabdomyosarcoma (RMS) | 3 | 1 | 2 |
|  | Rhabdoid tumour | 3 | 0 | 3 |
|  | Lipoblastoma | 3 | 0 | 3 |
|  | Angiomatoid fibrous histocytoma | 2 | 0 | 2 |
|  | Desmoid fibromatosis | 2 | 0 | 2 |
|  | Alveolar soft-part Sarcoma (ASPS) | 1 | 0 | 1 |
|  | Dermatofibrosarcoma protuberans (DFSP) | 1 | 0 | 1 |
|  | Synovial sarcoma | 1 | 0 | 1 |
|  | Benign spindle cell lesion | 1 | 0 | 1 |
|  |  |  |  |  |

Supplementary Table 3 : Known cancer-associated genes with autosomal dominant or/and autosomal recessive inheritance interrogated in whole exome data of this study.

| **Gene name** | **Inheritance pattern** |
| --- | --- |
| *ALK* | Autosomal dominant |
| *APC* | Autosomal dominant |
| *ATM* | Autosomal dominant, Autosomal recessive |
| *ATR* | Autosomal dominant, Autosomal recessive |
| *AXIN2* | Autosomal dominant |
| *BAP1* | Autosomal dominant |
| *BARD1* | Autosomal dominant |
| *BLM* | Autosomal recessive |
| *BMPR1A* | Autosomal dominant |
| *BRAF* | Autosomal dominant |
| *BRCA1* | Autosomal dominant |
| *BRCA2* | Autosomal dominant, Autosomal recessive |
| *BRIP1* | Autosomal dominant, Autosomal recessive |
| *CBL* | Autosomal dominant |
| *CDC73* | Autosomal dominant |
| *CDH1* | Autosomal dominant |
| *CDK4* | Autosomal dominant |
| *CDKN1C* | Autosomal dominant |
| *CDKN2A* | Autosomal dominant |
| *DDB2* | Autosomal dominant |
| *DICER1* | Autosomal dominant |
| *DIS3L2* | Autosomal recessive |
| *EPCAM* | Autosomal dominant |
| *ERCC2* | Autosomal recessive |
| *ERCC3* | Autosomal recessive |
| *ERCC4* | Autosomal recessive |
| *ERCC5* | Autosomal recessive |
| *FANCA* | Autosomal recessive |
| *FANCB* | Autosomal recessive |
| *FANCC* | Autosomal recessive |
| *FANCD2* | Autosomal recessive |
| *FANCE* | Autosomal recessive |
| *FANCF* | Autosomal recessive |
| *FANCG* | Autosomal recessive |
| *FANCI* | Autosomal recessive |
| *FANCL* | Autosomal recessive |
| *FANCM* | Autosomal recessive |
| *FH* | Autosomal dominant, Autosomal recessive |
| *GPC3* | X-linked recessive |
| *HRAS* | Autosomal dominant |
| *KRAS* | Autosomal dominant |
| *MAP2K1* | Autosomal dominant |
| *MAP2K2* | Autosomal dominant |
| *MAX* | Autosomal dominant |
| *MEN1* | Autosomal dominant |
| *MITF* | Autosomal dominant, Autosomal recessive |
| *MLH1* | Autosomal dominant, Autosomal recessive |
| *MRE11A* | Autosomal recessive |
| *MSH2* | Autosomal dominant, Autosomal recessive |
| *MSH6* | Autosomal dominant, Autosomal recessive |
| *MUTYH* | Autosomal recessive |
| *NBN* | Autosomal recessive |
| *NF1* | Autosomal dominant |
| *NF2* | Autosomal dominant |
| *NRAS* | Autosomal dominant |
| *NTHL1* | Autosomal recessive |
| *PALB2* | Autosomal dominant, Autosomal recessive |
| *PCNA* | Autosomal recessive |
| *PHOX2B* | Autosomal dominant |
| *PMS2* | Autosomal dominant, Autosomal recessive |
| *POLE* | Autosomal dominant, Autosomal recessive |
| *PRKAR1A* | Autosomal dominant |
| *PTCH1* | Autosomal dominant |
| *PTEN* | Autosomal dominant |
| *PTPN11* | Autosomal dominant |
| *RAD50* | Autosomal recessive |
| *RAD51* | Autosomal dominant |
| *RAD51C* | Autosomal dominant, Autosomal recessive |
| *RAD51D* | Autosomal dominant |
| *RAD54L* | Autosomal dominant |
| *RAF1* | Autosomal dominant |
| *RB1* | Autosomal dominant |
| *RECQL4* | Autosomal recessive |
| *RET* | Autosomal dominant |
| *RIT1* | Autosomal dominant |
| *SDHA* | Autosomal dominant, Autosomal recessive |
| *SDHAF2* | Autosomal dominant |
| *SDHB* | Autosomal dominant |
| *SDHC* | Autosomal dominant |
| *SDHD* | Autosomal dominant |
| *SHOC2* | Autosomal dominant |
| *SLX4* | Autosomal recessive |
| *SMAD4* | Autosomal dominant |
| *SMARCA4* | Autosomal dominant |
| *SMARCB1* | Autosomal dominant |
| *SMARCE1* | Autosomal dominant |
| *SOS1* | Autosomal dominant |
| *SOS2* | Autosomal dominant |
| *STK11* | Autosomal dominant |
| *SUFU* | Autosomal dominant |
| *TMEM127* | Autosomal dominant |
| *TP53* | Autosomal dominant |
| *TSC1* | Autosomal dominant |
| *TSC2* | Autosomal dominant |
| *VHL* | Autosomal dominant |
| *WRN* | Autosomal recessive |
| *WT1* | Autosomal dominant |
| *XPA* | Autosomal recessive |
| *XPC* | Autosomal recessive |
| *XRCC3* | Autosomal dominant |

Supplementary Table 4 : 29 known cancer-associated genes investigated by digital MLPA in this study.

| **Gene name** | **Inheritance pattern** |
| --- | --- |
| *APC* | Autosomal dominant |
| *ATM* | Autosomal dominant, Autosomal recessive |
| *BAP1* | Autosomal dominant |
| *BARD1* | Autosomal dominant |
| *BMPR1A* | Autosomal dominant |
| *BRCA1* | Autosomal dominant |
| *BRCA2* | Autosomal dominant, Autosomal recessive |
| *BRIP1* | Autosomal dominant, Autosomal recessive |
| *CDKN2A* | Autosomal dominant |
| *CDK4* | Autosomal dominant |
| *CDH1* | Autosomal dominant |
| *CHEK2* | Autosomal dominant |
| *EPCAM* | Autosomal dominant |
| *GREM1/SCG5* | Autosomal dominant |
| *MITF* | Autosomal dominant, Autosomal recessive |
| *MLH1* | Autosomal dominant, Autosomal recessive |
| *MSH2* | Autosomal dominant, Autosomal recessive |
| *MSH6* | Autosomal dominant, Autosomal recessive |
| *MUTYH* | Autosomal recessive |
| *NBN* | Autosomal recessive |
| *PALB2* | Autosomal dominant, Autosomal recessive |
| *PMS2* | Autosomal dominant, Autosomal recessive |
| *POLE* | Autosomal dominant, Autosomal recessive |
| *PTEN* | Autosomal dominant |
| *RAD51C* | Autosomal dominant, Autosomal recessive |
| *RAD51D* | Autosomal dominant |
| *SMAD4* | Autosomal dominant |
| *STK11* | Autosomal dominant |
| *TP53* | Autosomal dominant |

Supplementary Table 5 : Variants of uncertain significance (VUS) identified in this study listed by gene in alphabetical order.

| **Gene** | **Mutation type** | **DNA change (hg19)** | **Protein change** | **Sample ID** |
| --- | --- | --- | --- | --- |
| *ALK* | Missense | c.3476A>T | p.Gln1159Leu | DR378 |
| *ALK* | Missense | c.4208C>T | p.Pro1403Leu | DR615 |
| *ALK* | Missense | c.2210C>T | p.Ser737Leu | DR594 |
| *ALK* | Missense | c.3664G>C | p.Ala1222Pro | DR680 |
| *ALK* | Missense | c.386G>T | p.Gly129Val | DR594 |
| *ALK* | Missense | c.4307G>A | p.Arg1436His | DR614 |
| *ATM* | Missense | c.8246A>T | p.Lys2749Ile | DR383 |
| *ATM* | Missense | c.275A>C | p.Lys92Thr | DR603 |
| *ATM* | Missense | c.3295G>A | p.Asp1099Asn | DR369 |
| *ATR* | Missense | c.6833C>A | p.Ala2278Asp | DR394 |
| *ATR* | Missense | c.5740C>T | p.Pro1914Ser | DR608 |
| *ATR* | Missense | c.1898A>G | p.Gln633Arg | DR678 |
| *AXIN2* | Missense | c.1931A>G | p.Tyr644Cys | DR646 |
| *BLM* | Missense | c.2515A>G | p.Lys839Glu | DR683 |
| *BLM* | Missense | c.4165A>G | p.Thr1389Ala | DR676 |
| *BRCA1* | Missense | c.533T>A | p.Val178Asp | DR642 |
| *BRCA2* | Missense | c.8524C>T | p.Arg2842Cys | D147 |
| *BRCA2* | Missense | c.2573G>C | p.Arg858Thr | DR594 |
| *BRIP1* | Missense | c.1361A>T | p.Glu454Val | DR598 |
| *CBL* | Missense | c.2260A>G | p.Asn754Asp | DR612 |
| *CBL* | Missense | c.2155G>T | p.Ala719Ser | DR680 |
| *CDH1* | Missense | c.1118C>T | p.Pro373Leu | DR557 |
| *CDKN1C* | Nonframeshift deletion | c.600_611del | p.200_204del | DR655 |
| *CDKN1C* | Nonframeshift insertion | c.632_633insACCGGC | p.Ala211delins | DR605 |
| *CDKN2A* | Missense | c.344T>A | p.Val115Glu | DR614 |
| *DICER1* | Missense | c.4195A>G | p.Lys1399Glu | DR639 |
| *DIS3L2* | Missense | c.880A>G | p.Lys294Glu | DR617 |
| *DIS3L2* | Missense | c.871G>C | p.Ala291Pro | DR690 |
| *HRAS* | Missense | c.430A>G | p.Thr144Ala | D189 |
| *MAX* | Missense | c.298C>T | p.Arg100Cys | DR551 |
| *MLH1* | Missense | c.776T>C | p.Leu259Ser | DR614 |
| *MLH1* | Missense | c.29G>T | p.Arg10Leu | DR684 |
| *MLH1* | Missense | c.1136A>C | p.Tyr379Ser | DR550 |
| *MSH6* | Missense | c.3996A>T | p.Leu1332Phe | DR593 |
| *MSH6* | Missense | c.2483T>C | p.Val828Ala | DR602 |
| *MSH6* | Missense | c.1701G>C | p.Lys567Asn | DR627 |
| *NBN* | Missense | c.911C>T | p.Pro304Leu | DR601 |
| *NF2* | Missense | c.977G>C | p.Arg326Thr | DR612 |
| *PALB2* | Missense | c.3428T>A | p.Leu1143His | DR647 |
| *PALB2* | Missense | c.2435C>T | p.Pro812Leu | DR389 |
| *PMS2* | Missense | c.230A>C | p.Glu77Ala | DR608 |
| *PTCH1* | Missense | c.1285G>A | p.Asp429Asn | DR676 |
| *PTCH1* | Missense | c.2105C>G | p.Pro702Arg | DR672 |
| *PTCH1* | Missense | c.2072C>T | p.Thr691Ile | DR676 |
| *PTCH1* | Missense | c.3913G>A | p.Asp1305Asn | DR389 |
| *PTCH1* | Missense | c.110G>A | p.Cys37Tyr | DR624 |
| *PTCH1* | Nonframeshift deletion | c.142_144del | p.48_48del | DR685 |
| *PTPN11* | Missense | c.392A>G | p.Lys131Arg | DR596 |
| *RAD54L* | Missense | c.973G>A | p.Gly325Arg | DR676 |
| *RAD54L* | Missense | c.1976G>A | p.Gly659Asp | DR599 |
| *RAD54L* | Missense | c.2063G>A | p.Arg688His | DR549 |
| *RAF1* | Missense | c.994C>G | p.Pro332Ala | DR617 |
| *RB1* | Missense | c.524C>T | p.Thr175Ile | DR688 |
| *RECQL4* | Missense | c.1685G>A | p.Arg562Gln | DR370 |
| *RECQL4* | Missense | c.3172C>T | p.Arg1058Cys | DR679 |
| *RECQL4* | Missense | c.2099T>C | p.Leu700Pro | D147 |
| *RECQL4* | Missense | c.1444C>T | p.Arg482Cys | DR685 |
| *RECQL4* | Missense | c.2928G>C | p.Glu976Asp | DR602 |
| *RECQL4* | Nonframeshift insertion | c.2574_2575insTGCACCTGCACC | p.Arg859delins | DR650 |
| *RET* | Missense | c.1597G>A | p.Gly533Ser | DR376 |
| *RET* | Missense | c.1597G>A | p.Gly533Ser | DR639 |
| *RET* | Missense | c.1597G>A | p.Gly533Ser | DR685 |
| *RET* | Missense | c.2776C>T | p.His926Tyr | DR621 |
| *RET* | Missense | c.1438G>A | p.Glu480Lys | DR548 |
| *RET* | Missense | c.1891G>A | p.Asp631Asn | DR644 |
| *RET* | Missense | c.624G>T | p.Glu208Asp | D147 |
| *RET* | Missense | c.1295C>A | p.Ala432Glu | DR672 |
| *SDHA* | Missense | c.1591G>A | p.Val531Met | DR608 |
| *SDHA* | Missense | c.739A>G | p.Ile247Val | DR646 |
| *SMARCA4* | Missense | c.1018G>A | p.Ala340Thr | DR652 |
| *SMARCE1* | Missense | c.484T>A | p.Ser162Thr | DR370 |
| *SOS2* | Missense | c.3250A>G | p.Thr1084Ala | DR646 |
| *SOS2* | Missense | c.20C>G | p.Pro7Arg | DR653 |
| *SOS2* | Missense | c.2290A>G | p.Ser764Gly | DR616 |
| *STK11* | Missense | c.1127A>C | p.Glu376Ala | DR612 |
| *SUFU* | Missense | c.169A>G | p.Ile57Val | D147 |
| *TSC1* | Missense | c.2485A>C | p.Ser829Arg | DR622 |
| *TSC1* | Missense | c.1827G>C | p.Glu609Asp | DR685 |
| *TSC1* | Missense | c.3266G>C | p.Gly1089Ala | DR394 |
| *TSC2* | Missense | c.1676A>T | p.Asp559Val | DR690 |
| *TSC2* | Missense | c.1241G>C | p.Cys414Ser | DR555 |
| *TSC2* | Nonframeshift deletion | c.4025_4027del | p.1342_1343del | DR645 |
| *WRN* | Missense | c.2197C>T | p.Pro733Ser | DR549 |
| *WRN* | Missense | c.2098G>A | p.Val700Ile | DR651 |
| *WT1* | Missense | c.749T>A | p.Met250Lys | DR379 |
| *WT1* | Missense | c.1547C>A | p.Ala516Glu | DR595 |

**SUPPLEMENTARY FIGURES**

Supplementary Figure 1 : qPCR validation of *TP53* exon 1 deletion detected by MLPA in blood and tumour-derived DNA of patient LGG08-0816 clearly demonstrated hemizygous germline deletion and homozygous deletion in the tumour. Pt: Patient


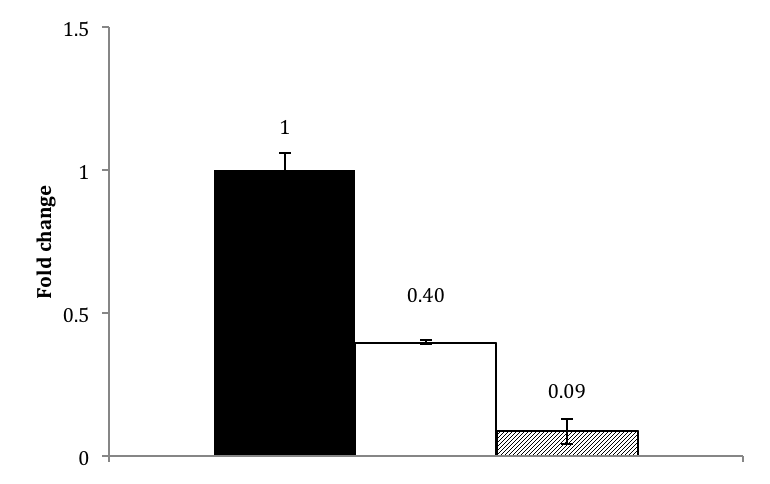


Healthy (n=2)

Pt blood

Pt tumour

Supplementary Figure 2: Distribution of VUS by tumour type, frequency is normalized by incidence of tumour type within the cohort.
